# Synthesis and application of stationary phase for DNA-affinity chromatographic analysis of unmodified and antisense oligonucleotide

**DOI:** 10.1007/s00216-021-03473-7

**Published:** 2021-06-24

**Authors:** Sylwia Studzińska, Ewelina Zawadzka, Szymon Bocian, Michał Szumski

**Affiliations:** 1 Chair of Environmental Chemistry and Bioanalytics, Faculty of Chemistry, Nicolaus Copernicus University in Toruń, 7 Gagarin St., 87-100 Toruń, Poland; 2 Centre for Modern Interdisciplinary Technologies, Nicolaus Copernicus University in Toruń, Wileńska 4, 87-100 Toruń, Poland

**Keywords:** Oligonucleotides, DNA-affinity chromatography, Oligonucleotide-based stationary phase, Selectivity, Retention, Liquid chromatography

## Abstract

The goal of the research was the synthesis and application of an oligonucleotide immobilized stationary phase for the analysis of unmodified and antisense oligonucleotides. The method for attaching these molecules to aminopropyl silica modified with pentanedioic acid was developed. Each step of the synthesis was carefully controlled with the application of spectroscopic, elemental, and chromatographic analyses. The oligonucleotide-based stationary phase was applied for the retention studies. Unmodified oligonucleotides of different complementarity to the molecule attached as a stationary phase, as well as antisense oligonucleotides, were tested. The comparative study upon complex optimization of oligonucleotide analysis in different liquid chromatography modes was performed. Results have shown that this stationary phase may be applied for oligonucleotide analysis in hydrophilic interaction liquid chromatography and ion exchange chromatography, but no unique sequence-based selectivity was obtained. Contrary results were observed for affinity chromatography, which allowed for specific separation of the complementary strands based on hydrogen bonding and stacking interactions, where the temperature was the main factor influencing the selectivity of the separation. Furthermore, the oligonucleotide-based stationary phase may be applied for comparative antisense oligonucleotide hybridization studies to a specific RNA sequence. All of the results have shown that affinity chromatography with oligonucleotide-based stationary phases is a powerful technique for the specific base recognition of polynucleotides.

**Graphical abstract:**

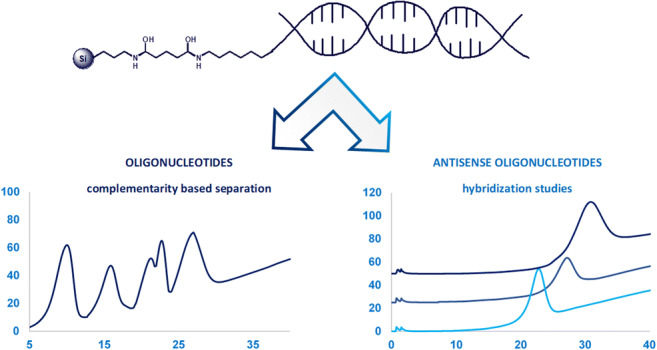

**Supplementary Information:**

The online version contains supplementary material available at 10.1007/s00216-021-03473-7.

## Introduction

Affinity chromatography (AC) is used generally for the purification of complementary biological molecules that can interact specifically with each other. The application of AC to nucleic acids and oligonucleotides (OGNs) is not common yet, although the first DNA-silica chromatographic column was presented as far as in 1990 [1]. The DNA-affinity chromatography (DNA-AC) is applied for selective fractionation of nucleic acids and OGNs, their purification and separation [2–4]. The retention of these analytes results from selective and reversible interactions (electrostatic interactions and/or hydrogen bonding between complementary base pairs) between analyte and stationary phase ligands (OGNs, DNA, or RNA fragments) bound to the solid surface [2]. There are many supports to which OGN can be bounded, such as silica gel, agarose, organic polymers, gold plates, magnetic nanoparticles, or quantum dots [2–9]. Such materials are used for the hybridization of nucleic acids or OGNs and applied as biosensors, extraction supports, or even drug transporters [2–10]. Despite the application of various supports, silica gel is still the most common one used for DNA-affinity chromatography due to the widely developed ways of its modification. Various strategies have been used for immobilizing OGNs at the surface of solid supports over the years. The procedures for coupling 5′-aminoalkyl-OGN to silica activated with, e.g., N-hydroxysuccinimide, diisothiocyanate, and dicarboxylic polyethylene glycol have been described [4, 6, 11]. In contrast, also 5′-terminal carboxylic acid-derivatized oligodeoxyribonucleotides may be chemically bound to aliphatic amino groups at the modified silica surface [4, 6, 11]. The synthesis of the DNA-affinity chromatography stationary phase by the formation of an amide bond is a simple and effective method (it proceeds in one step under mild conditions and leads to stable products) [6, 11]. However, enzymatic synthesis of DNA-silica has been also reported: enzymatic synthesis of biotinylated DNA and its binding on streptavidin support. This methodology is more selective and prevents nucleotide bases from being unfavorably modified as it would be during the chemical methods [12]. The DNA-AC is typically used for purification of DNA-binding proteins, transcription factors, enzymes, and polynucleotides as well as studying interactions of DNA with other molecules [4, 13–15]. Despite the fact that the DNA-AC technique is capable of isolating and identifying target molecules complementary for a specific ligand, it is rarely used for separation based typically just on DNA hybridization, e.g., when specific sequences present in DNA are recognized by an immobilized OGN forming a helix. It takes place in the case of plasmids or mRNA purification methods [16, 17]. For example, 20-mer deoxythymine coupled to solid support hybridized to the poly-adenylated tail in mRNAs, while contaminant impurities (proteins, nucleotides, plasmid DNA, double-strand RNA, enzymes) were not retained at such a stationary phase surface [17]. This process is characterized by very high selectivity and could be particularly interesting for the isolation of target OGNs from complex biological extracts. For this reason, one of the possible fields of DNA-AC application may be the isolation of modified, antisense oligonucleotides (ASO), which are widely used as inhibitors of gene expression or protein synthesis [18]. These OGNs used in antisense therapy are synthetic molecules with modifications performed in the structure of a phosphate group, a sugar, or a nucleobase. After their introduction to the cell, they interact and bind to the complementary fragment of DNA or RNA [18, 19]. There is a necessity for the development of ASO selective separation methods, and DNA-AC could be particularly interesting due to hybridization effects [18]. There has been just one attempt described in the scientific literature presenting its application to the fractionation of phosphorothioate oligonucleotides [20]. DNA-AC appeared to be useful in the separation of diastereoisomers of this group of modified OGNs [20].

The main aim of the present study was the synthesis of an OGN immobilized silica gel column and its application for the study of unmodified and antisense OGNs. The method for attaching OGNs to silica modified with pentanedioic acid was developed, and each step of the synthesis was carefully controlled using FTIR spectroscopy, and elemental and chromatographic analysis. Next, the column was applied to study the retention and separation of OGNs of different complementarity to the molecule bonded to the support surface. The present work describes a comparative study upon complex optimization of OGN analysis in three different liquid chromatography modes.

## Materials and methods

### Materials

OGN standards were purchased in a lyophilized form from Sigma-Aldrich (Gillingham, Dorset, UK) and Eurogentec (Seraing, Belgium). Their sequences, molecular masses, and modifications are presented in Table 1. Ten-micromolar solutions of OGNs were prepared by dilution with deionized water.

**Table 1:** Sequences and molecular masses of studied oligonucleotides

Abbreviation	Number of nucleotides in sequence	Sequence 5′-3′	Molecular mass[g mol^−1^]
DNA	20	TGACGGATGCCAGCTTGGGC-(CH_2_)_7_-NH_2_	6288
OL1	20	GCCCAAGCTGGCATCCGTCA	6063
OL2	20	GCCCAAGCTGGCAT**GGCAG**A	6152
OL3	20	G**GGG**AAGCTGGCATCCGTCA	6183
OL4	20	GCCCAAGCT**AA**CATCCGTCA	6031
OL5	20	GCCC**TT**GCTGGCATCCGTCA	6045
OL6	16	AAAAAAAAAAAAAAAA	4949
OL7	20	ATCGATCGATCGATCGATCC	6077
OL8	15	ATTTTTTTTTTTTTT	4510
OL9	20	ATTGGAACCTTAAGGCCCAA	6110
PS	20	G*C*C*C*A*A*G*C*T*G*G*C*A*T*C*C*G*T*C*A	6368
ME	20	GmCmCmCmAmAmGmCmTmGmGmCmAmTmCmCmGmTmCmA	6621

* phosphorothioate modification, *m* 2′-*O*-alkyl substitution

The following reagents were used for the synthesis: 1-ethyl-3-(3-dimethylaminopropyl)carbodiimide (EDC), morpholineethanesulfonic acid (MES), sodium citrate, sodium chloride, pentanedioic acid, γ-aminopropyltrimethoxysilane, and toluene (all purchased from Sigma-Aldrich, Gillingham, Dorset, UK). The Kromasil 300 silica gel, with particle diameter of 5 μm, pore diameter of 300 Å, pore volume of *V*_*p*_ = 0.9 mL/g, and surface area *S*_*BET*_ = 110 m^2^/g (Akzo Nobel, Bohus, Sweden), was used as the support for the synthesis.

Mobile phases were prepared from methanol (Sigma-Aldrich, Gillingham, Dorset, UK) and deionized water (Milli-Q system, Millipore, El Paso, TX, USA). Mobile phase additives such as sodium chloride, sodium perchlorate monohydrate, ammonium acetate, sodium citrate, magnesium chloride, and Tris-HCl (all analytical grade) were purchased from Sigma-Aldrich (Gillingham, Dorset, UK).

### Apparatus and chromatographic conditions

The Thermo Scientific™ Vanquish™ Horizon UHPLC system equipped with a diode array detector (Thermo Scientific, CA, USA) and controlled with Chromeleon 7 chromatography data software was used during the research. The ACE Excel C18Ar (1.7 μm, 100 × 2.1 mm) column purchased from Advanced Chromatography Technologies Ltd. (Aberdeen, UK) was applied during the estimation of DNA immobilization efficiency. The chromatographic experiments were performed with a mobile phase composed of methanol (MeOH) and 5 mM ammonium acetate using a 5–25% v/v of MeOH during a 6-min gradient elution program.

In the case of the SG-DNA stationary phase, the mobile phase composition was related to the liquid chromatography mode used during the studies. The mixture of 10 or 100 mM of ammonium acetate and methanol was applied for hydrophilic interaction liquid chromatography (HILIC). Solutions of 0.1 or 0.5 M of sodium chloride or sodium perchlorate were used for ion exchange chromatography (IEC). In the case of AC, mobile phases were composed of sodium chloride (from 0.025 to 0.5 M), sodium citrate or Tris-HCl (from 0.0025 to 0.05 M), or MgCl_2_ (from 10 to 20 mM). During all experiments, the mobile phase flow rate was set as 0.3 mL min^−1^. The injection volume was 1 μL, and the detection was performed at the wavelength of λ = 260 nm. Different column temperatures were applied ranging between 10 and 90 °C.

FTIR spectra were recorded using a FTIR Vertex 70 V Bruker Optik spectrophotometer (Bruker Corporation, Billerica, MA, USA). Spectra were taken in the ranges of 400–4000 cm^−1^ and 1300–4000 cm^−1^. Due to low carbon content in the investigated material, spectra were averaged over 6000 scans. The carbon and nitrogen percentages were determined using a Model 240 CHN analyzer (Perkin Elmer, Norwalk, USA).

The samples were centrifuged with a 5424-μL Eppendorf AG centrifuge (Hamburg, Germany) and a CentriVap vacuum concentrator (Labconco, Kansas City, MO, USA). A heating-cooling dry block (Grant Instruments Ltd., Royston, Great Britain) and a laboratory pH meter (Elmetron CP-505, Zabrze, Poland) were also used.

### Synthesis of the SG-DNA stationary phase

**Fig. 1:**
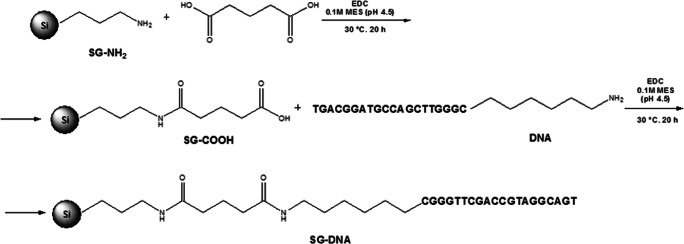
Scheme of the SG-DNA synthesis procedure based on the aminopropyl (SG-NH_2_) stationary phase modification with pentanedioic acid (SG-COOH) followed by oligonucleotide molecule (DNA) immobilization

During this research, the aminopropyl-modified silica (SG-NH_2_) adsorbent was used as a support for further modifications. It was synthesized in the Chair of Environmental Chemistry and Bioanalytics, according to the procedure described earlier in ref. [21]. In the first step, the SG-NH_2_ surface was modified with pentanedioic acid. To do that, 300 mg of EDC and 36 mg of pentanedioic acid dissolved in 1 mL of 0.1 M MES solution (pH 4.5) were added to 500 mg of SG-NH_2_ placed in an Eppendorf vial and mixed. The synthesis was performed in a heating block at 30 °C for 20 h. Next, the suspension was centrifuged (15 min, 14,000 rpm) and washed three times with 0.1 M MES (pH 4.5) and once with PBS solution. The obtained SG-COOH material was dried and used in the final step of synthesis in which the aminoalkyl-modified DNA (Table 1) was immobilized at the adsorbent surface. For this purpose, 300 mg of SG-COOH and 600 mg EDC were dissolved in 750 μL of 0.1 M MES solution (pH 4.5) and such a mixture was composed of 3660 μL of 100 μM DNA solution (also dissolved in 0.1 M MES, pH 4.5). The amount of DNA used for the synthesis was selected based on our earlier studies [22]. The synthesis was performed by placing the vial in a heating block at 30 °C for 20 h. After the reaction, the suspension was centrifuged (15 min, 14,000 rpm) and washed with the mixture of 0.5 M NaCl and 0.05 M sodium citrate, followed by PBS solution. After washing, the SG-DNA product was dried. The scheme of SG-DNA stationary phase synthesis is presented in Fig. 1.

SG-DNA stationary phase was packed into 125 mm × 2.1 mm I.D. stainless-steel columns using a high-pressure LC-column packing station consisting of the Haskel DSF-122 air-amplified pump (Burbank, CA, USA) and slurry reservoirs with a precolumn and connectors fitting the column internal diameter. The stationary phase slurry was prepared in mixtures of different solvents and sonicated for 20 min before packing (see in “Results and discussion”). Acetonitrile or methanol was used as packing liquids. Columns were packed at a constant pressure of 40 MPa for 1 h.

## Results and discussion

### DNA immobilization on the silica surface

The method applied for pentanedioic acid and DNA immobilization at the SG-NH_2_ turned out to be simple and effective and was based on a carbodiimide-mediated acylation in the acidic pH of MES buffer (to obtain the most efficient crosslinking) [11].

**Fig. 2:**
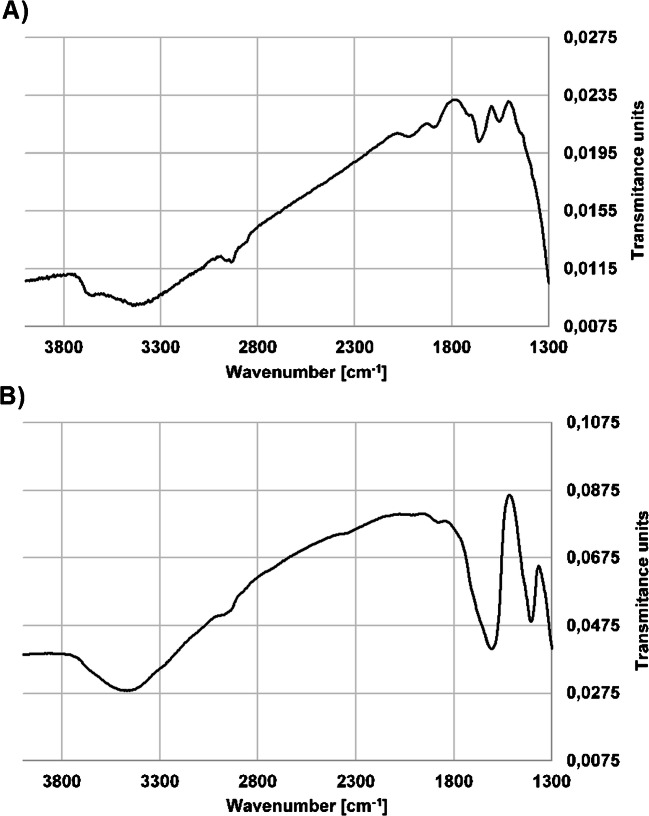
FTIR spectra (KBr) of the synthesized materials: **A** SG-COOH, **B** SG-DNA

Each material obtained and used during the synthesis (SG-NH_2_, SG-COOH, SG-DNA) was investigated using FTIR spectroscopy in order to confirm the bonding of pentanedioic acid and DNA molecules to the silica surface. The comparison of spectra taken with SG-COOH and a final product (SG-DNA) is presented in Fig. 2 and Fig. S1 in the Supplementary information (ESM). In both cases, high-intensity bands characteristic for the silica gel structure are present: asymmetrical bending vibrations Si-O-Si at 476 cm^−1^ and symmetrical stretching for Si-O at 812 cm^−1^ (ESM Fig. S1).

On the other hand, the wide band in the range 3400–3550 cm^−1^ observed for SG-COOH is characteristic for the stretching vibrations of the –OH group of carboxylic acid, as well as residual silanols at the silica surface. The band with a maximum at 1660 cm^−1^ corresponds to the stretching vibrations of the group C=O (Fig. 2A). Moreover, a signal at 1550 cm^−1^ corresponds to carboxylates (Fig. 2A). All of these signals confirm the presence of free carboxyl groups at the surface of the support. In the case of SG-DNA, a strong signal in the range of 3300–3600 cm^−1^ reflects the stretching vibrations of the –O–H and –N–H groups, as well as the stretching vibrations of the –O–H group for deoxyribose (3500 cm^−1^) and –N–H groups characteristic for nitrogen bases (3600 cm^−1^) (Fig. 2B). The intensity of this signal is greater when compared to SG-COOH. After the modification of SG-COOH with DNA molecules, additional signals were observed which confirmed the presence of nitrogen-hydrogen and nitrogen-carbon bonds present in OGNs structure. These are bands of C–N stretching vibration at around 1400 cm^−1^ and N–H bend at 1600 cm^−1^ (Fig. 2B). The presence of signals corresponding to –C–H, –C–N (1400 cm^−1^), and –C–OH in sugars (3500–3300 cm^−1^), as well as –O–H in deoxyribose (3330 cm^−1^) and –N–H groups (1600–1550 cm^−1^ and 3500 cm^−1^) characteristic for nitrogen bases confirmed the successful immobilization of DNA molecules at the silica gel surface.

It has to be emphasized here that the efficiency of the DNA immobilization was assessed by HPLC determination of the amount of DNA remaining in the solution after the synthesis and each washing step. Based on the obtained results, it was concluded that none of the DNA was released into the solution during the washing step. Moreover, all DNA used for the synthesis was bonded to the carboxylic groups at the SG-COOH surface.

Elemental analysis of SG-NH_2_ and materials synthesized during the study (SG-COOH and SG-DNA) revealed that for SG-NH_2_ nitrogen and carbon contents were 0.47 and 1.51%, respectively, while in the case of SG-COOH, the amount of carbon increased to 4.16%, while nitrogen remained more or less on the same level that is 0.51%. These values confirm the successful modification of aminopropyl ligands with pentanodioic acid. The elemental analysis also confirmed the successful modification of SG-COOH with DNA, because both carbon and nitrogen content increased (5.99% of carbon and 0.78% of nitrogen). The calculated coverage density was 1.1 nmol/m^2^. Relatively low surface coverage in comparison with traditional stationary phases for liquid chromatography is caused by two factors: (1) large bonded ligand (oligonucleotide) and (2) free space between the bonded ligands necessary for analyte interaction basis on complementarity with bonded ligands. Higher surface coverage may hamper the interaction with an analyte basis on complementarity and reduce the selectivity of the material synthesized.

The SG-DNA material is not a typical HPLC material, so following initial experiments regarding the stability of the stationary phase, slurries, three different conditions were chosen: (1) the slurry was prepared in 2-propanol and methanol was used for packing, (2) the slurry was prepared in 2-propanol/methanol/water mixture (2:2:1 v/v/v) and acetonitrile was used for packing, and (3) the slurry was prepared in the 2-propanol/methanol mixture (10:3 v/v) and acetonitrile was applied for packing. There was also an attempt to pack the column with 0.1 M NaCl solution. Despite different packing conditions, each time the column efficiency was achieved at approximately 3000 theoretical plates per 125 mm column. The column efficiency turned out to be rather poor. Here, two reasons could be attributed to this fact. On one hand, we are aware that the column packing methodology should be improved in further more complex and systematic studies and this was restricted by the costs in the present research. On the other hand, however, broad peaks are likely the result of the complex nature of a relatively great number of interactions between the analytes and SG-DNA stationary phase. Moreover, the stationary phase surface inhomogeneity and probably low DNA coverage density as well as the interactions between the residual amine and carboxyl groups could play some role.

### Chromatographic analysis of OGNs with the use of SG-DNA

The purpose of the SG-DNA synthesis was its application for selective analysis of OGNs differing with the degree of complementarity to the DNA OGN. Therefore, OGNs that were characterized by full (OL1) or partial (OL2–OL5) complementarity, as well as those that are not complementary (OL6–OL10) to DNA were selected for the study. Details of OL1–OL5 are listed in Table 1. Chromatographic analysis of OGNs of different sequences allowed investigating to what extent the retention of the analytes was affected by (1) the formation of complementary base pairs and degree of hybridization, (2) electrostatic interactions, and (3) sequence length and molecular mass of OGNs.

Three different liquid chromatography modes were used during the study in order to check the possible applicability of SG-DNA for the OGN analysis under different mobile phase compositions.

#### Hydrophilic interaction liquid chromatography

The mobile phases used during this step of studies included mixtures of methanol with 10 mM or 100 mM of ammonium acetate. The influence of salt concentration, methanol percentage, and temperature on the retention of tested compounds was investigated. Despite the changing of all of these parameters, the elution of complementary OGNs was not possible under applied conditions; therefore, only retention data for the other, non-complementary compounds were presented in Table S1 (see ESM).

It was surprising that the *k* values increased with the increase of methanol content in the mobile phase, contrary to effects commonly observed in RP HPLC (ESM Table S1). The lowest *k* values were observed for the mobile phase with the lowest methanol content. Such trend of retention over the mobile phase composition suggests that the retention mechanism was more similar to HILIC than to RP HPLC, despite the application of MeOH instead of ACN. This conclusion is also supported by the property of the stationary phase that is relatively polar. OGNs may be deprived the hydration shell, and consequently, their gradual denaturation leads to an increased affinity to the stationary phase surface.

The *k* values also increased when salt concentration increased from 10 to 100 mM (ESM Table S1). This indicates that the elution strength of the mobile phase decreases with increasing salt concentration, which caused an increase in the affinity of the analyte for the stationary phase. Salt ions screen the electrostatic repulsion between the negatively charged backbones and allow interaction between the bonded DNA and analyzed OGN. It results also from the “salting-out” effect.

It should be noted that SG-DNA was washed with water after finishing each step of the study. Complementary OGNs were then eluted from the column.

#### Ion exchange chromatography

The 0.1 M and 0.5 M solutions of NaCl or NaClO_4_ were used as IC mobile phases. The gradient elution was used starting with 100% v/v 0.1 M salt solution and ending with 100% v/v 0.5 M of salt within 15 min. Preliminary analyses were performed for all OGNs: complementary and non-complementary ones. Unfortunately, under these chromatographic conditions, OGNs complementary to DNA (OL1, OL4, OL5) were not eluted from the SG-DNA column that is on another hand confirmation of highly selective adsorption. Therefore, further IC studies were carried out for non-complementary OGNs and results were presented in Table S1 (see ESM). Lower *k* values were obtained for NaClO_4_, which indicates greater elution strength of this salt compared to NaCl. On the other hand, even when NaCl was used, the OGN retention was low (ESM Table S1). It should be emphasized that the charge on the surface of the stationary phase and in the structure of the analyzed compounds is negative, so retention is a result of hydrogen and electrostatic bonds and van der Waals forces that increase with the analyte size. On the other hand, high salt concentration provides sodium adducts to each phosphate group in OGN structure. Consequently, the electrostatic repulsion is reduced and retention becomes greater.

#### Affinity chromatography

The elution conditions in AC are typically selected by changing the pH, ionic strength, or polarity of the eluent, as well as by the addition of compounds that break hydrogen bonds. In AC, the sample is usually washed out from the column using a high salt concentration or a buffer. Salt ions screen the electrostatic repulsion between the negatively charged backbones of DNA and analyzed OGNs; consequently, they allow for the interactions between the stationary phase and the analyte. For this reason, three different mobile phases were tested: NaCl in sodium citrate (pH 7); NaCl in TRIS-HCl (pH 7); and NaCl, sodium citrate, and MgCl_2_ (pH 7). This step of the study was performed in a gradient mobile phase composition changing from 100% v/v of salt to 100% v/v of water or in a temperature gradient. Such a complex study for OGNs of different complementarity degree was performed for the first time. Table 2 shows the results obtained for OL1, OL2, and OL4 as well as OL6 and OL7. The sodium citrate and TRIS-HCl buffers were used to maintain a constant pH of the mobile phase. The *k* values were similar when sodium citrate or TRIS-HCl were applied, indicating their low impact on OGN retention (Table 2). In contrast, the addition of MgCl_2_ was aimed at reducing electrostatic repulsion between negatively charged phosphate groups of OGNs. The base-pairing interactions are stabilized additionally by lateral forces between the helical parts of the OGNs occurring at a high Mg^2+^ concentration. Therefore, increasing OGN *k* values were observed when MgCl_2_ was added to the mobile phase. Moreover, retention was greater when MgCl_2_ concentration increased (Table 2). However, it should be noticed that peak symmetry was worsened.

**Table 2:** The impact of the mobile phase composition on retention factor values for OGNs analyzed in AC. Experimental conditions: temperature gradient from 10 to 80 °C in 5 min, next the temperature was constant at 80 °C; for other conditions, see in the “Materials and methods” section

Oligonucleotide	*k* at different mobile phases						
	0.025 M NaCl in 2.5 mM sodium citrate (pH = 7)	0.05 M NaCl in 5 mM sodium citrate (pH = 7)	0.1 M NaCl in 10 mM sodium citrate (pH = 7)	0.15 M NaCl in 15 mM sodium citrate (pH = 7)	0.1 M NaCl in 10 mM TRIS-HCl (pH = 7)	0.05 M NaCl in 5 mM sodium citrate, 5 mM MgCl_2_ (pH = 7)	0.05 M NaCl in 5 mM sodium citrate, 10 mM MgCl_2_ (pH = 7)
OL1	t_M_	25.7 ± 0.6	43.5 ± 0.6	Not eluted	42.9 ± 0.7	35.7 ± 0.7	39.2 ± 0.5
OL2	t_M_	17.0 ± 0.3	26.6 ± 0.1	34.6 ± 0.5	25.6 ± 0.1	22.3 ± 1.0	25.00 ± 0.4
OL4	t_M_	14.6 ± 0.4	20.7 ± 0.2	25.2 ± 0.6	25.0 ± 0.3	17.9 ± 0.4	21.0 ± 0.4
OL6	t_M_	t_M_	0.8 ± 0.08	1.1 ± 0.06	0.9 ± 0.06	0.6 ± 0.08	0.8 ± 0.07
OL7	t_M_	t_M_	0.8 ± 0.07	1.0 ± 0.1	0.9 ± 0.04	0.5 ± 0.1	0.8 ± 0.03
OL8	t_M_	t_M_	0.4 ± 0.04	0.9 ± 0.08	0.8 ± 0.01	t_M_	0.4 ± 0.1

The effect of NaCl concentration (in a mixture with sodium citrate) on OGN retention under the temperature gradient elution conditions from 10 to 80 °C was also studied. Four salt concentrations were tested: 0.025 M, 0.05 M, 0.1 M, and 0.15 M. Results are presented in Table 2. The tendency to increase OGN retention with increasing salt concentration was observed. These compounds were not retained at the SG-DNA surface when very low (25 mM) salt concentration was used as mobile phase, indicating its important role in the interactions between OGNs and stationary phase surface. As it was summarized earlier, Na^+^ cations shield the electrostatic repulsion between the negatively charged backbones of analyzed OGNs, as well as OGNs bonded to silica surface. Moreover the “salting-out” effect probably also occurs causing a greater affinity of OGNs to SG-DNA. For these two reasons, increasing the concentration of these ions in the mobile phase resulted in greater *k* values. The significant *k* value difference was noticed for both complementary OGNs and non-complementary ones (Table 2). The second group of the tested compounds (OL6, OL7, OL8) was retained on SG-DNA stationary phase to a lesser extent (or they were not retained at all when 25 or 50 mM of salt was used) compared to complementary OGNs, and the *k* values were very similar (Table 2). This effect proves SG-DNA selectivity towards sequences of high or medium complementarity degree to the molecule bonded to the support surface. In the case of non-complementary OGNs, their retention on the surface of SG-DNA is the result of the “salting-out” effect. The retention of complementary OGNs, on the other hand, is a result of the screening of negatively charged phosphate groups by Na^+^ ions and hydrogen bonds between complementary purine and pyrimidine base pairs. We could easily separate non-complementary OGNs from complementary OL1, OL2, and OL4 just by applying proper salt concentration (Table 2). The SG-DNA may be applied for the fractioning of OGNs with those of low complementarity being eluted in the dead volume, or close to the dead volume. Simultaneously with increasing salt concentration, the analytes’ peaks were more symmetrical compared to the mobile phases containing just 0.05 M NaCl.

The elution of complementary OGNs in AC occurs usually under a low concentration of buffer or under water mobile phase. Therefore, the electrostatic repulsion between the negatively charged backbones of immobilized DNA and OGNs destabilizes the nucleobase pairs and releases OGNs from the column. Another factor affecting OGN retention in AC is the temperature. Raising the temperature destabilizes the double helix formed of two complementary OGNs which leads to its melting or denaturation (breaking of hydrogen bonds between complementary nucleobases) which, in consequence, can significantly affect the retention of the oligonucleotides. Further studies were then focused on both temperature and mobile phase composition change.

**Fig. 3:**
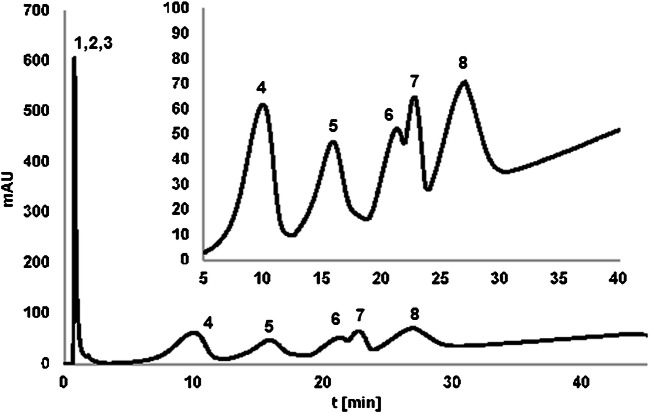
Chromatograms of the separation of the oligonucleotide mixture on the SG-DNA column in AC. Experimental conditions: mobile phase composition: 0.05 M NaCl, 0.005 M sodium citrate; flow rate 0.3 mL/min; autosampler temperature 4 °C; UV-Vis detection at λ = 260 nm; column temperature: 0 min—10 °C, 2.5 min—10 °C, 10 min—90 °C, 45 min—90 °C. Analytes: 1—OL6, 2—OL7, 3—OL9, 4—OL2, 5—OL3, 6—OL4, 7—OL5, 8—OL1

Regarding the temperature, the retention studies were performed in the isocratic elution mode at different temperatures (T changed stepwise from 80 to 10 °C in 10° steps). All of the studied OGNs were eluted in the dead volume at the highest temperature, while at 70 °C only OL1 and OL2 were retained at the SG-DNA surface. OL4 and OL3 were retained by such a stationary phase when the lower temperature was applied (60 °C). It may be concluded that with the increase of complementarity degree, higher temperature is needed in order to elute OGNs from the column. It stays true for non-complementary OL6, OL7, and OL8 for which lowering the temperature to 10 °C was needed. These findings were then used for the separation of the mixture of the studied compounds in the temperature-governed elution. Figure 3 presents the example chromatogram of OGN mixture separation. It can be seen that according to the above observations, OGN elution strongly depended on the degree of complementarity of analyzed compounds to the molecule bonded to the silica surface. The non-complementary OGNs were eluted in the first fractions. All other OGNs complementary to the attached ligand were eluted later as only their interactions with SG-DNA underwent heat denaturation. OL2 differing with 5 nucleotides in the sequence was eluted as the first one, whereas OL3 (differing with 3 nucleotides) had greater retention. OL4 and OL5 were not separated to the baseline, because they have similar sequences with differences in the position of just two nucleotides. OL1 had the greatest retention, because of its completely complementary sequence to the bound DNA molecule. Such separation of OGNs differing just in the type and position of single nucleotides was successfully performed for the first time. Chromatographic behavior was then easily predictable in AC using such a stationary phase and the elution order of OGNs could be simply controlled by designing an appropriate sequence of the immobilized molecule. As OGNs naturally form double-stranded complexes due to the presence of the specific interactions (complementary hydrogen bonding and stacking interactions), the development of a stationary phase in which one strand of OGN is immobilized on support allows the specific separation of the complementary strand in mixtures of polynucleotides. However, the successful separation was possible due to the temperature changes, contrary to results obtained when water was used as the eluent of the highest elution strength.

**Fig. 4:**
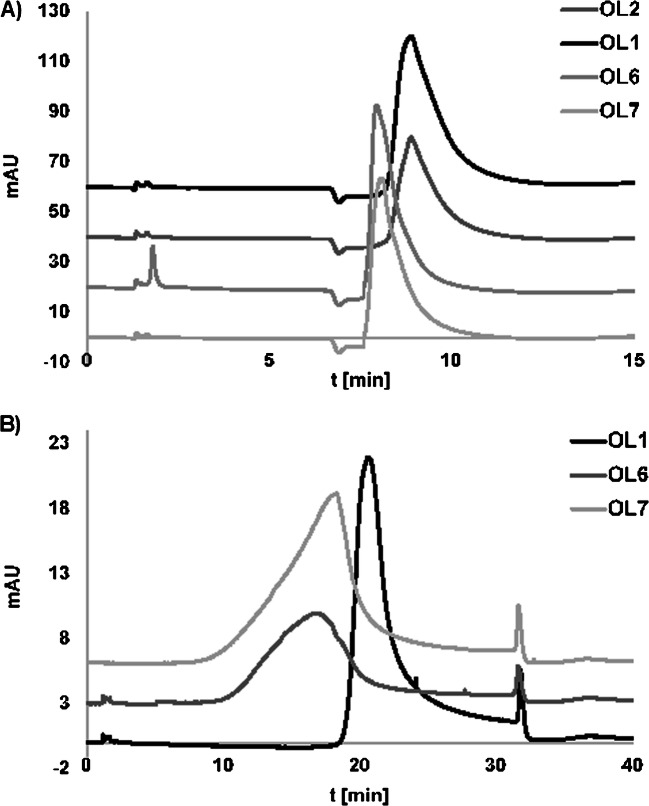
Chromatograms of OGNs separated on the SG-DNA column at different experimental conditions: **A** mobile phase composition: 0.5 M sodium chloride/0.05 M sodium citrate and water, gradient elution program: 0–5 min 100% of salt solution to 100% of water at 5.1 min, then 5.1–50 min 100% of water; **B** mobile phase composition 0.05 M sodium chloride/0.005 M sodium citrate and water, gradient elution program: 0 min—100% of salt solution, 20 min—0% of salt (100% of water), 20–30 min—100% of water. Other conditions: flow rate 0.3 mL/min, UV-Vis detection at λ = 260 nm, column temperature 20 °C, injection volume 2 μL

The application of stepwise gradient elution of 0.5 M NaCl in 0.05 M sodium citrate and water at 20 °C caused similar retention of studied OGNs (e.g., OL6 7.9 min, OL1 8.87 min) (Fig. 4A). Both complementary and non-complementary ones were eluted with water. However, greater differences in the retention were noticed when the salt concentration was reduced (to 0.05 M) and a shallow gradient was used. The examples of the observed trends are presented in Fig. 4B. There are slight differences in retention times and peak symmetry for OGNs which are non-complementary to the OGN bonded with the stationary phase surface (Fig. 4B). Complementary OGNs probably form duplexes with OGNs at SG-DNA, contrary to non-complementary OGNs in which case their interaction with the surface of the stationary phase through the hydrogen bonds of nucleobases is weaker. For this reason, they are eluted from the chromatographic column earlier, and the shape of the peaks is asymmetric.

In our opinion, the above results showed that heating denaturation provided better results of OGN separation compared to changing the elution strength of the mobile phase. The presented method may be easily applied for OGN purification. More efficient OGN purification procedures are needed not only for routine laboratory handling of synthesis samples but also for the development of the selective ASOs analysis method. For this reason, we have attempted to study the retention of ASO OGNs with sequences complementary to DNA, but different modification types: phosphorothioate PS (at each phosphate group) and 2′-O-methyl ME (at each ribose molecule) (Table 1). The main goal of these modifications is to improve the biostability and cellular uptake of OGNs. On the other hand, modified ASOs must maintain the hybridization properties of unmodified OGNs so they could bind to the RNA fragment. This type of study was done for the first time in DNA-AC for ASOs.

**Fig. 5:**
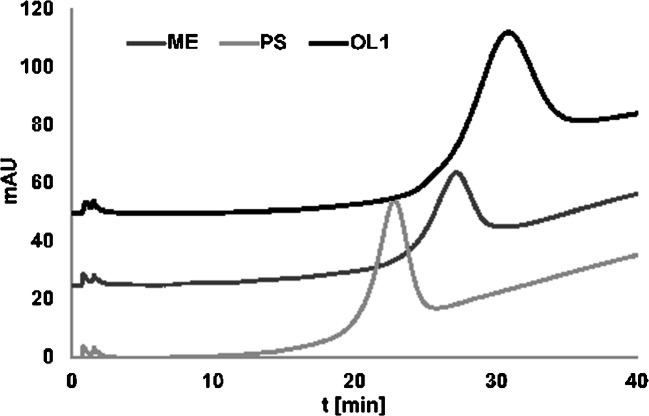
Example chromatograms of unmodified (DNA1) and modified (PS, ME) OGN analysis with the use of an SG-DNA column. Experimental conditions: mobile phase composition—0.05 M NaCl, 0.005 M sodium citrate; flow rate 0.3 mL/min; autosampler temperature 4 °C; UV-Vis detection λ = 260 nm; column temperature: 10–80 °C in 20 min

The examples of the results obtained during this step of our study are presented in Fig. 5. Both PS and ME OGNs were retained at the SG-DNA surface but their retention was weaker compared to OL1. Moreover, ME formed more stable hybrids with DNA compared to PS. These findings are in accordance with the literature data [23, 24]. The phosphorothioates had a comparably low binding capacity to the complementary nucleic acids, while 2′-*O*-alkyl substitutions stabilized the duplex [23, 24]. In recent years a variety of modified ASOs are examined for their ability to bind specifically to a target RNA. Results presented in the current study indicate that AC may be a good analytical tool for the comparative study of ASO hybridization with target RNA sequences.

## Conclusions

DNA-affinity chromatography is a powerful technique for the specific base recognition of OGNs, but its utilization is developing slowly due to the low durability of packing materials. However, the synthesis method presented in this study is based on the application of pentanedioic acid and DNA immobilization on the aminopropyl stationary phase appeared to be simple and reliable allowing effective OGN bonding at the support surface.

OGNs of different complementarity to the molecule immobilized at the stationary phase were studied and it was shown that the sequence has a great impact on their retention on SG-DNA. The use of HILIC or IEC made it possible to retain non-complementary OGNs on the surface of the stationary phase, but nevertheless, it was not possible to wash out the complementary OGNs. These compounds were eluted during flushing the column with water. These effects have shown that SG-DNA may be applied for OGN analysis in HILIC or IEC, but no unique sequence-based selectivity will be obtained. Contrary results were observed for AC. Application of buffered NaCl as mobile phase together with a temperature gradient allowed for the first time for a specific separation based on differences in complementarity and stacking interactions. The temperature was the main factor influencing the selective separation of OGNs in AC during the present study. The developed method may be successfully applied for OGN purification, for separation of failure sequences, and for the study of OGNs differing with complementarity degree. Furthermore, it may find many other applications. On the other hand, successful separation of complementary and non-complementary OGN mixture was not possible under applied conditions. However, this result may be an advantage, because quick fractionation of OGNs based on complementarity is possible.

The main advantage of the presented results is successful application of SG-DNA to the ASO study. This column may be applied for ASO hybridization studies. The importance of hybridization was shown in the literature by its correlation with antisense activity observed in cell assays. It was for the first time presented that liquid chromatography with oligonucleotide-based stationary phases may be a simple tool for comparative studies of ASO hybridization to a specific RNA sequence. These results are very promising and studies will be extended.

## Supplementary information

ESM 1 (DOCX 816 kb)

## References

[1] Goss T, Bard M, Jarrett H (1990). High-performance affinity chromatography of DNA. J Chromatogr A.

[2] Hage DS, Cazes J (2005). Handbook of affinity chromatography.

[3] Mitra S, Moxley RA, Jarrett H, Celis JE. Chapter 43 - DNA affinity chromatography of transcription factors: the oligonucleotide trapping approach, cell biology (third edition). Academic Press. 2006:335–42.

[4] Chockalingam PS, Jurado LA, Jarret HW (2001). DNA affinity chromatography. Mol Biotechnol.

[5] Gadgil H, Jarrett HW (2002). Oligonucleotide trapping method for purification of transcription factors. J Chromatogr A.

[6] Lund V, Schmid R, Rickwood D, Hornes E (1998). Assessment of methods for covalent binding of nucleic acids to magnetic beads, Dynabeads, and the characteristics of the bound nucleic acids in hybridization reactions. Nucleic Acids Res.

[7] Balamurugan S, Obubuafo A, Soper SA, McCarley RL, Spivak DA (2006). Designing highly specific biosensing surfaces using aptamer monolayers on gold. Langmuir.

[8] Tikhomirov G, Hoogland S, Lee PE, Fischer A, Sargent EH, Kelley SO (2011). DNA-based programming of quantum dot valency, self-assembly and luminescence. Nat Nanotechnol.

[9] Zhang CY, Yeh HC, Kuroki MT, Wang TH (2005). Single-quantum-dot-based DNA nanosensor. Nat Mater.

[10] Jo H, Ban C (2016). Aptamer–nanoparticle complexes as powerful diagnostic and therapeutic tools. Exp Mol Med.

[11] Walsh MK, Wang X, Weimer BC (2001). Optimizing the immobilization of single-stranded DNA onto glass beads. J Biochem Biophys Methods.

[12] Solomon L, Massom L, Jarrett HW (1992). Enzymatic syntheses of DNA-Silicas using DNA polymerase. Anal Biochem.

[13] Jarrett HW (2000). Temperature dependence of DNA affinity chromatography of transcription factors. Anal Biochem.

[14] Locke IC, Cox SF, Carpenter BG (1997). Purification of a streptococcal deoxyribonuclease by affinity chromatography based on a DNA-cellulose matrix. J Chromatogr A.

[15] Corthesy PB, Kao PN (1994). Purification by DNA affinity chromatography of two polypeptides that contact the NF-AT DNA binding site in the interleukin 2 promoter. J Biol Chem.

[16] Sousa F, Prazeres DMF, Queiroz J (2008). Affinity chromatography approaches to overcome the challenges of purifying plasmid DNA. Trends Biotechnol.

[17] Goss TA, Brad M, Jarrett HW (1991). High-performance affinity chromatography of messenger RNA. J Chromatogr A.

[18] Studzińska S (2018). Review on investigations of antisense oligonucleotides with the use of mass spectrometry. Talanta.

[19] Skoblov MY (2009). Prospects of antisense therapy technologies. Mol Biol.

[20] Zarytova VF, Shishkina IG (1990). Affinity chromatography of DNA fragments and P-modified oligonucleotides. Anal Biochem.

[21] Buszewski B, Gadzala-Kopiuch RM, Jaroniec M (1997). Chromatographic properties of mixed chemically bonded phases with alkylamide and aminopropyl ligands. J Liq Chromatigr Rel Technol.

[22] Studzińska S, Skoczylas M, Bocian S, Dembska A, Buszewski B (2020). Attachment of hybridizable oligonucleotide to silica support and its application for selective extraction of unmodified and antisense oligonucleotides from serum samples. RSC Adv.

[23] Freier SM, Altmann KH (1997). The ups and downs of nucleic acid duplex stability: structure-stability studies on chemically-modified DNA:RNA duplexes. Nucleic Acids Res.

[24] Kurreck JK, Wyszko E, Gillen C, Erdmann VA (2002). Design of antisense oligonucleotides stabilized by locked nucleic acids. Nucleic Acids Res.

