# Supplementary material for: Synthesis and application of stationary phase for DNA-affinity chromatographic analysis of unmodified and antisense oligonucleotide
Source: Anal Bioanal Chem. 2021 Jun 24;413(20):5109–19. doi: 10.1007/s00216-021-03473-7 (PMC8405468; doi:10.1007/s00216-021-03473-7)
Supplement: Supplementary file 1 — (DOCX 816 kb) [file 216_2021_3473_MOESM1_ESM.docx]

**Supplementary Information**

**Synthesis and application of stationary phase for DNA affinity chromatographic analysis of unmodified and antisense oligonucleotide**

Sylwia Studzińska^*^, Ewelina Zawadzka, Szymon Bocian, Michał Szumski

Chair of Environmental Chemistry and Bioanalytics, Faculty of Chemistry, Nicolaus Copernicus University in Toruń,7 Gagarin St., 87-100 Toruń, Poland

*Corresponding author, e-mail: [kowalska@chem.umk.pl](mailto:kowalska@chem.umk.pl)

**B)**

**A)**

**Figure S1** FTIR spectra (KBr) of the synthesized materials in the whole range (400-4000 cm^-1^): A) SG-COOH, B) SG-DNA.

**Table S1** Retention data obtained for oligonucleotides analysed with the use of SG-DNA under HILIC and IC conditions

| Oligonucleotides | *k* | | | |
| --- | --- | --- | --- | --- |
|  | HILIC | | | |
|  | 5% v/v MeOH | 5% v/v MeOH | 20% v/v MeOH | 35% v/v MeOH |
|  | 95 % v/v 10 mM CH_3_COONH_4_ | 95 % v/v 100 mM CH_3_COONH_4_ | 80 % v/v 100 mM CH_3_COONH_4_ | 65 % v/v 100 mM CH_3_COONH_4_ |
| OL6 | 0.1±0.01 | 0.9±0.09 | 3.2±0.2 | 10.0±0.3 |
| OL7 | 0.1±0.02 | 5.00±0.12 | 9.7±0.1 | 17.3±0.3 |
| OL8 | 0.1±0.02 | 2.3±0.05 | 4.8±0.2 | 10.9±0.4 |
|  | IC | | | |
|  | 0.1-0.5 M NaCl in 15 min | | 0.1-0.5 M NaClO_4_ in 15 min | |
| OL6 | 0.9±0.2 | | t_M_ | |
| OL7 | 0.8±0.1 | | t_M_ | |
| OL8 | 0.5±0.08 | | 0.3±0.09 | |
